# Digital Information Exchange Between the Public and Researchers in Health Studies: Scoping Review

**DOI:** 10.2196/63373

**Published:** 2025-01-28

**Authors:** Nazli Soltani, Thilo Dietz, Doris Ochterbeck, Jens Dierkes, Katja Restel, Lara Christianson, Karina Karolina De Santis, Hajo Zeeb

**Affiliations:** 1 Department of Prevention and Evaluation Leibniz Institute for Prevention Research and Epidemiology – BIPS Bremen Germany; 2 Institute of Medical Sociology, Health Services Research, and Rehabilitation Science (IMVR) University of Cologne Cologne Germany; 3 Department of Research and Publication Support University and City Library University of Cologne Cologne Germany; 4 Faculty of Human and Health Sciences University of Bremen Bremen Germany

**Keywords:** health information, information exchange, communication, knowledge translation, dissemination, digital technology, research participant, scoping review

## Abstract

**Background:**

Information exchange regarding the scope and content of health studies is becoming increasingly important. Digital methods, including study websites, can facilitate such an exchange.

**Objective:**

This scoping review aimed to describe how digital information exchange occurs between the public and researchers in health studies.

**Methods:**

This scoping review was prospectively registered and adheres to the PRISMA-ScR (Preferred Reporting Items for Systematic Reviews and Meta-Analyses Extension for Scoping Reviews) guidelines. Eligibility was defined using the population (public and researchers), concept (digital information exchange), and context (health studies) framework. Bibliographic databases (MEDLINE, PsycINFO, CINAHL, and Web of Science), bibliographies of the included studies, and Google Scholar were searched up to February 2024. Studies published in peer-reviewed journals were screened for inclusion based on the title, abstract, and full text. Data items charted from studies included bibliographic and PCC (Population, Concept, and Context) characteristics. Data were processed into categories that inductively emerged from the data and were synthesized into main themes using descriptive statistics.

**Results:**

Overall, 4072 records were screened, and 18 studies published between 2010 and 2021 were included. All studies evaluated or assessed the preferences for digital information exchange. The target populations included the public (mainly adults with any or specific diseases), researchers, or both. The digital information exchange methods included websites, emails, forums, platforms, social media, and portals. Interactivity (ie, if digital information exchange is or should be active or passive) was addressed in half of the studies. Exchange content included health information or data with the aim to inform, recruit, link, or gather innovative research ideas from participants in health studies. We identified 7 facilitators and 9 barriers to digital information exchange. The main facilitators were the consideration of any stakeholder perspectives and needs to clarify expectations and responsibilities, the use of modern or low-cost communication technologies and public-oriented language, and continuous communication of the health study process. The main barriers were that information exchange was not planned or not feasible due to inadequate resources, highly complex technical language was used, and ethical concerns (eg, breach of anonymity if study participants are brought together) were raised. Evidence gaps indicate that new studies should assess the methods and the receiver (ie, public) preferences and needs that are required to deliver and facilitate interactive digital information exchange.

**Conclusions:**

Few studies addressing digital information exchange in health studies could be identified in this review. There was little focus on interactivity in such an exchange. Digital information exchange was associated with more barriers than facilitators, suggesting that more effort is required to improve such an exchange between the public and researchers. Future studies should investigate interactive digital methods and the receiver preferences and needs required for such an exchange.

## Introduction

### Public Engagement With Health Research

Public engagement with research can improve the visibility, quality, and impact of such research. The term “public engagement” is used to describe the various ways in which the public (ie, the general population) can be involved in the design, conduct, and dissemination of research. It may be employed to inform and inspire, to advise and listen, or to collaborate with the public. Engagement is by definition a 2-way process involving interaction and listening and intending to generate mutual benefit [1]. A conceptual review of 142 peer-reviewed articles published between 1996 and 2019 showed that the term “public engagement” converges with the term “public involvement.” This is supported by the increasing use of the combination term “patient and public involvement and engagement” [2].

In the context of health research with human participants, the public (ie, the general population) should have a say on how to safely participate in studies and how the data collected in such studies is used [3]. Integrating public engagement in the whole research process includes sharing research with nonacademic audiences by contributing to dissemination plans and materials for the public and supporting the use of research in practice [4]. It has been suggested that enhanced involvement of the public (eg, study participants or other interested laypeople) can improve executive procedures such as recruitment and data collection [5,6]. For clinical trials, information exchange, including bidirectional sharing of information, such as data and feedback, between researchers and the public creates a more satisfying trial experience for all involved (ie, study participants and researchers) [7]. The dissemination of study results is defined as one of the key aspects of patient and public involvement and engagement in health research, where patients should provide input on the communication strategy of the study and be co-authors of study publications [8]. A systematic review on youth involvement in health research identified several benefits of such involvement, including that it provides unique perspectives that help to improve research questions and priorities and that it leads to improved translation, dissemination, and uptake of research findings [9].

Furthermore, incorporating the perspectives of the public in research right from the beginning can help researchers to identify and focus on issues relevant to the target group. This might not only improve the proximity of research to practice and thus enhance its translation, but also increase the acceptance and willingness to participate in health studies [1,8]*.* A mixed-methods study using quantitative surveys and qualitative interviews with researchers and patients assessed patient experiences and perceptions of engagement in health studies [10]. Survey results identified key factors influencing engagement, such as communication quality and participant expectations, highlighting areas for improved collaboration. Interviews provided deeper insights into patient experiences, emphasizing the importance of mutual respect, transparency, and addressing barriers, including unclear roles or logistical challenges of communication. These findings suggest that well-structured frameworks are needed to plan meaningful patient involvement that is required to enhance the effectiveness of patient-researcher partnerships [10].

### Digital Information Exchange

Owing to the great advancements in technology during the past decades and the increased accessibility of technological tools and devices, health information exchange is now possible with less effort and expense than before. It can thus help transform the role of study participants from passive observers and subjects in research studies to active members of study teams, who can have an impact on the conduct and outcome of a research study. For example, citizen scientists (participants in a study on arthritis) recorded their daily pain intensity in a smartphone app [11]. The GPS location of their phones was then linked to local weather data to look for possible associations between weather conditions and pain intensity. It was thus possible to collect large datasets and develop a tool to assist patients in better managing their respective health condition [11]. However, there are various barriers associated with the use of digital methods for health information exchange, including poor digital health literacy, ethical and legal issues, low trust in online information sources, and poor technological affinity. For example, in the context of COVID-19, it was difficult for the public to obtain appropriate and accurate online information due to uncertainty about the disease and a plethora of inconsistent content [12]. When it comes to the dissemination of research results, the complexity of the research process and the use of appropriate plain rather than technical language are problematic. Well-designed websites built in association with medical professionals, which have a clear interface and contain quality health information, can draw the attention of patients and lead them to access trustworthy information [13]. Having broad access to additional health information over the internet, which patients can use for discussions with health care professionals, can lead to more patient involvement and engagement in medical decision-making [14].

To develop and establish participation and information exchange opportunities using digital methods, it is vital to explore what is available and, in particular, if these methods are accepted by the target group, that is, the public, including study participants and any laypeople. The goal of such participatory research is to assess the wants and needs of all stakeholders involved in health research.

### Digital Portals for Health Information Exchange

The lexical definition of a portal describes a door, passageway, or entrance area that allows entry into a large or grand building, thus providing access to a new separate area [15]. This meaning can also be applied to digital infrastructure. In this context, an internet-based digital portal aggregates information from numerous sources and makes it accessible to a diverse group of users [16]. Thus, digital portals offer an opportunity for digital information exchange in health research.

The early literature distinguishes between different types of health portals [17] that provide access to a range of functions [18]. The first developments of patient portals for access to personal health information can be traced back to the 1990s [19-21]; however, their more widespread use started later in about 2006 [22]. The definitions of the term “patient portal” and its functions vary. A patient portal refers to an application provided by a health care institution (eg, a hospital) that allows patients to access general health information [23]. However, a patient portal could also provide secure access to the patient’s electronic health record (EHR) [20,24-26]. Studies indicate the benefits of patient portals, as they can enhance doctor-patient communication [27] and link patient information with other information on the internet in the case of digital portals [23]. Further studies indicate other benefits in terms of active patient participation and increased satisfaction and treatment adherence [28-31]. The use of a patient portal depends on the expected benefit to the user and the cognitive resources that need to be invested to use it. On average, users are younger, chronically ill, and able to understand digital health information compared to patients who do not use such portals but are aware of their existence [32].

The onset of the COVID-19 pandemic was associated with the increased availability of “open health data.” This type of data can originate from public authorities (eg, health authorities), clinical records (eg, EHR), or public health–related surveys [33]. The increasing availability of these data has been accompanied by the development and organization of digital networks and portals for data exchange. These digital portals allow, in part, unrestricted, free, and unlimited access to open health data in machine-readable format for all interested parties [33]. While patients may be interested in the development and progression of a disease, authorities, governments, and medical institutions may need information on the spread of diseases [34]. To meet these demands, there is an increasing need for sophisticated and user-oriented digital portals with health data [33].

According to nonsystematic searches of the internet via Google, we identified 5 examples of digital portals with health data (Table 1). These digital portals are available in German, English, or multiple languages. The target population of the portals included either the public or researchers. Overall, 4 portals focused on data exchange, while 1 portal allowed patients to search for health information using a filter option.

**Table 1:** Examples of digital health portals.

Portal name	Targeted population	Portal aim	Portal focus	Search terms used to locate the portal in Google
Gesund.bund.de: Verlässliche Informationen für Ihre Gesundheit [35]	Patients, family members, care takers, and the public (interested parties)	Detailed search for information on diseases and the provision of assistance in the event of a disease	Information exchange	“Patientenportal” OR “patient information portal”
European Health Information Portal [36]	Researchers	Search for existing study records; data exchange	Data exchange	“Patientenportal” OR “patient information portal”
Health Data Lab [37]	Researchers and legislators	Access and exchange of health and care data	Data exchange	“health study data” OR “health data portal”
HealthData.gov [38]	Researchers, entrepreneurs, and legislators	Identification of trial and health data	Data exchange	“health study data” OR “health data portal”
Add Health – The National Longitudinal Study of Adolescent to Adult Health/Add Health Navigator [38,39]	Public (participants and interested parties) and researchers	Access study data by searching for records using the “Add Health Navigator”; study-specific portal for data sharing and exchange	Data exchange	“health study data” OR “health data portal”

### NFDI4Health (The National Research Data Infrastructure for Personal Health Data) Initiative in Germany

The National Research Data Infrastructure for Personal Health Data (NFDI4Health) is one example of a nationwide digital health portal established in Germany that has the overarching goal of providing new opportunities for the scientific use of personal health data while respecting privacy requirements. The central element of NFDI4Health is the operationalization of the FAIR (findable, accessible, interoperable, reusable) principles [40] around health data, especially those from epidemiological and clinical trials, to promote their reuse and strengthen the reputation of collaborating researchers. One important objective of NFDI4Health is the transfer of research results and study knowledge to the public, which should strengthen the interaction among the public, researchers, and health research institutions [41]. As part of this objective, a digital portal called Research Dialogue [42] has been developed to provide the public with online access to study information, results, and aggregated data from research projects listed in NFDI4Health. In the long term, this digital portal should enable an interactive (ie, bidirectional) exchange between the public and researchers to improve the understanding of health data and health research, ensure a low-threshold exchange of information, and promote participation in clinical studies in Germany. This study was designed to identify digital evidence-based methods from other studies that could be used in the development of the Research Dialogue portal for digital information exchange between the public and researchers.

### Study Aims and Objectives

This study aimed to describe how digital information exchange occurs between the public and researchers in health studies, using a scoping review methodology. A scoping review is particularly applicable when a body of scientific evidence needs to be described and mapped into categories without a focus on health outcomes. This review has the following 6 objectives:

1. Studies: What are the characteristics of assessments on digital information exchange between the public and researchers in health studies?
2. Population: What populations were addressed in these studies?
3. Concept: What methods of digital information exchange were described in these studies?
4. Context: What content and aim of digital information exchange were addressed in these studies?
5. Factors associated with digital information exchange: What facilitators and barriers associated with digital information exchange were identified in these studies?
6. Evidence gaps: What evidence gaps and ideas for future research were identified in these studies?

## Methods

### Study Design

This scoping review adheres to the PRISMA-ScR (Preferred Reporting Items for Systematic Reviews and Meta-Analyses Extension for Scoping Reviews) guidelines [43]. The PRISMA-ScR checklist is presented in Multimedia Appendix 1.

### Protocol and Registration

A protocol for this review was prospectively registered [44]. There were no changes between the protocol and the content of this review. At the time of protocol registration, preliminary searches were conducted to design the search strategy.

### Eligibility Criteria

The eligibility for this scoping review was based on the PCC (Population, Concept, and Context) framework (Textbox 1). The 3 components of this framework were defined as follows. “Population” includes the public that can include patients, study participants, or any laypeople (ie, the general population). This group is considered as the primary receivers of health information. “Population” can also include researchers (ie, any experts, such as academics, health care professionals, or other relevant stakeholders) who are primary generators and providers of health information (ie, they exchange or plan to exchange health information with the public). “Concept” includes the actual or planned health information exchange using any digital methods (eg, a website with health study content that can be accessed by the population). “Context” includes the health information from specific or any health studies.

**Eligibility criteria.**

**Inclusion criteria**

- Population: Public (ie, laypeople, general population, study participants, and patients) of any age and with any health status (ie, healthy, at risk for any disease, or with any disease) or researchers (eg, any experts, such as academics, health care professionals, or any relevant stakeholders) who exchange or plan to exchange information with the public
- Concept: Digital health information exchange or need for health information using any digital methods (eg, websites accessed using any devices, such as computers or smartphones)
- Context: Health study; (1) Focus on information exchange in a specific health study; (2) Focus on public or researcher perspectives on information exchange or need for health information in any health study
- Study design: Primary study with any design (eg, observational study or survey) or data type (quantitative or qualitative); reports on digital portals with health content
- Study type: Published in a peer-reviewed journal
- Language: English or German
- Access: Full text available

**Exclusion criteria**

- Population: (1) No focus on the public (eg, focus on information exchange among researchers, health care professionals, politicians, stakeholders, students, or other population groups); (2) Nonhuman populations (eg, veterinary health; meta-research studies on published articles or research methods)
- Concept: (1) Digital health information exchange in health care settings (eg, for clinical data collection or recording); (2) Nondigital health information exchange (eg, information exchange on paper or in face-to-face settings)
- Context: (1) No focus on health studies; (2) Focus on specific topics in the health context, including clinical treatment, online support groups, individual clinical data, electronic health records, and genome data; development of health systems, health frameworks, digital health technologies, clinical guidelines, procedures, or tools; marketing and sales of health products; ethical and legal issues in consent and data sharing; teaching, training, and health education for health care professionals; participant recruitment; health literacy; and quality of health information
- Study design: Nonprimary study (eg, literature review, comment, editorial, correction, or study protocol)
- Study type: Other study types (eg, conference papers, dissertations, or books)
- Language: Language other than English or German
- Access: Full text not available

### Information Sources

The information sources for this scoping review include 4 international bibliographic databases (MEDLINE, PsycINFO, CINAHL, and Web of Science), bibliographies of any included studies, and the internet (eg, Google Scholar).

### Search Strategy

The search syntax was developed by the team (NS and KKDS) with support from an experienced information specialist (LC). The search terms addressed the PCC criteria (Textbox 1) and included relevant synonyms and subject terms (Textbox 2). The search, using Boolean connectors, adjacency operators, and truncation, was designed in MEDLINE and adapted to other databases (Multimedia Appendix 2). The keyword search was limited to the title and abstract fields, and there were no language or other limits applied.

**Example search terms used in database searches. Population, concept, and context terms were combined with the Boolean operator “AND” in the search.**

**Population: public**

- Search terms: patient, subject, public, citizen, participant, lay, user, general population
- Subject terms: patients, “research subjects”

**Concept: digital information exchange**

- Search terms: digital or online health education, digital or online health information, digital or online health knowledge, health education or health information or health knowledge portal, health education or health information or health knowledge platform, health education or health information or health knowledge website, digital information exchange or communication or dissemination, online information exchange or communication or dissemination, information portal or information website or information platform access
- Subject terms: “health information exchange,” “consumer health information,” “information seeking behavior,” “information dissemination”

**Context: health study**

- Search terms: health research, health study, health trial, medical research, medical study, medical trial, epidemiological research, epidemiological study, epidemiological trial

The search was performed (by LC) from database inception to February 1, 2024. The search documentation is shown in Multimedia Appendix 2. All search results were exported to the Systematic Review Accelerator Deduplicator (Bond University Institute for Evidence-Based Healthcare) for deduplication and EndNote 20 (Clarivate) for management. Manual searches of bibliographies of the included studies and the internet via Google Scholar were performed (by NS, JD, and KR) up to February 2024. All search results were exported and managed in EndNote 20.

### Study Selection

Screening was performed in EndNote by 3 researchers (NS, KKDS, and a team assistant). Overall, 50% of all studies were screened by at least 2 researchers. As recommended by the PRISMA-ScR guidelines [43], the screening procedure was discussed in the team to increase the consistency among the screeners. The eligibility criteria (Textbox 1) were pilot tested on randomly selected studies (at least one study per criterion) until consensus was reached. Title and abstract screening was supported by the *smartgroups* function in EndNote that was used to identify studies for exclusion (eg, reviews, books, or conference papers). Two researchers (KKDS and a team assistant) checked and confirmed the exclusion of such studies identified by EndNote. Screening of all titles and abstracts was performed by 1 researcher (KKDS) and checked by another researcher (NS or a team assistant). Screening of full-text articles was performed by 2 researchers (NS and KKDS) independently, and final consensus was reached by discussion within the team (NS, KKDS, and a team assistant). Studies were included if they addressed any of the objectives of the review and if they provided details of any digital exchange methods or the intention to exchange the research findings with the public.

### Data Charting

A data charting form was developed and calibrated within the team. Data were charted by 2 researchers (NS and TD) who extracted author statements from the included studies and agreed on the final statement selection by discussion. The extracted data were processed into meaningful categories by 2 researchers (NS and TD), and a final consensus was reached by discussion with a third researcher (KKDS). Data processing was subsequently discussed within the team (NS, TD, and KKDS), and a consensus was reached by discussion.

Extracted data included quantitative information (eg, publication year) and qualitative information (eg, description of digital information exchange methods) per study. In scoping reviews, qualitative information is organized by categorizing data into main themes to provide structured insights [43]. In this scoping review, 2 researchers (NS and TD) read all extracted data and then classified them into meaningful categories that were inductively identified in the data. For example, we classified the methods of digital information exchange as “active” or “passive” based on the description of digital exchange extracted from each individual study. The categories were discussed within the team (NS, TD, and KKDS) and calibrated by discussion until a consensus was reached. The final categories were entered into the data charting form for each study for further analysis. The main themes were identified in the process of data synthesis.

### Data Items

A list of data items (Table 2) was developed by 1 researcher (NS) and discussed within the team (NS, TD, and KKDS).

**Table 2:** Data items based on the objectives of this scoping review.

Objective and data item		Data item content or definition
**Objective 1 (Studies: design and aim)**		
	1. Bibliographic characteristics	First author, title, publication year, corresponding author continent, funding source
	2. Study characteristics	Design (ie, primary study or report), aim (eg, evaluation or preferences for digital information)
**Objective 2 (population)**		
	3. Population characteristics	Type: Public (ie, laypeople, general population, study participants, and patients) who are primary receivers of digital health information or researchers (eg, any experts, such as academics, health care professionals, or any relevant stakeholders) who are primary providers of digital health information, age, location (continent), health focus (eg, specific disease or any disease)
**Objective 3 (concept: digital information exchange)**		
	4. Modality	Modality according to study authors (eg, website or other)
	5. Interactivity	Interactivity implicitly or explicitly described by study authors (ie, passive exchange or interactive exchange)
**Objective 4 (context: health study)**		
	6. Digital exchange content	Content (eg, data exchange, information exchange, or both); if data exchange, type of exchanged data (eg, aggregated study data or individual patient data, or both)
	7. Digital exchange aim	Aim (eg, to inform the public about the health study), format (eg, video or other)
**Objective 5 (factors associated with digital information exchange)**		
	8. Facilitators	Factors that could facilitate digital information exchange
	9. Barriers	Factors that could hinder digital information exchange
**Objective 6 (evidence gaps)**		
	10. Evidence gaps	Evidence gaps and ideas for future research with a focus on information exchange addressed in studies (eg, conclusion)

### Critical Appraisal

Critical appraisal was not performed because this scoping review aimed to describe central topics and questions related to digital information exchange rather than to evaluate the effectiveness of such exchange.

### Data Synthesis

The data processed into categories in each study were synthesized for all 18 studies using absolute and relative frequencies or means and SDs in Excel (version 10; Microsoft Corp) to address the 6 objectives of this scoping review. For example, 2 researchers (NS and TD) listed all categories relevant for objective 3 (methods of digital information exchange), counted all studies out of 18 that were assigned into each category, and noted their citations. The main themes in the data were identified by 3 researchers (NS, TD, and KKDS) by discussion. We read all individual categories assigned to each objective and clustered them based on their content. For example, we clustered the categories “active” or “passive” digital information exchange into a main theme “interactivity” as part of the methods of digital information exchange. For objectives 5 (factors associated with digital information exchange) and 6 (evidence gaps), we clustered the categories from individual studies into 3 main themes (ie, facilitators, barriers, and evidence gaps) and reported example author statements to further explain the meaning of each theme. These 3 themes were either explicitly mentioned by the study authors or were derived based on our interpretation of categories assigned to individual studies.

## Results

### Study Selection

**Figure 1:**
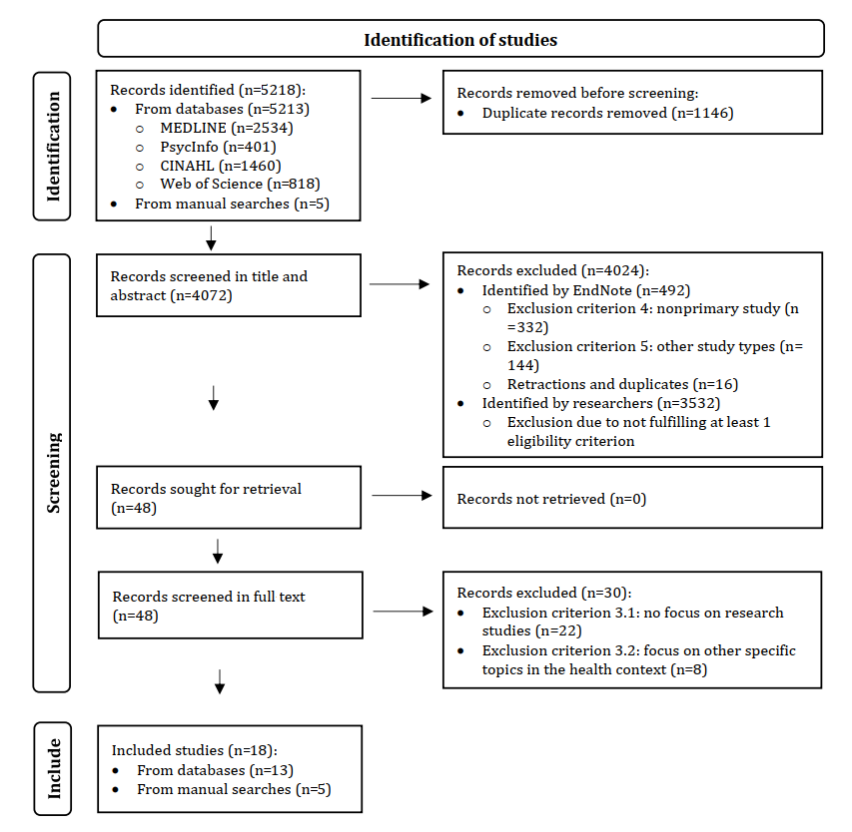
PRISMA-ScR (Preferred Reporting Items for Systematic Reviews and Meta-Analyses Extension for Scoping Reviews) flow diagram.

The electronic and manual searches identified 5218 records (Figure 1). Following the removal of duplicates, 4072 records were screened based on the title and abstract and 48 records were screened based on the full text. Overall, 492 of the 4072 records were selected for exclusion using EndNote (ie, nonprimary studies, including reviews and study protocols; other study types, including conference papers, dissertations, and books; and retracted articles or duplicates that were not detected by Deduplicator). Further, 3532 of the 4072 records were excluded as they did not fulfill at least 1 eligibility criterion (Textbox 1). The specific reasons for exclusion of these records were not documented, as suggested by the PRISMA-ScR guidelines [43]. Full-text screening identified 18 of 48 studies [7,45-61] that met the inclusion criteria and were included in this scoping review. The list of the 30 excluded studies with individual reasons for exclusion is presented in Multimedia Appendix 3.

### Bibliographic Characteristics of the Included Studies

**Figure 2:**
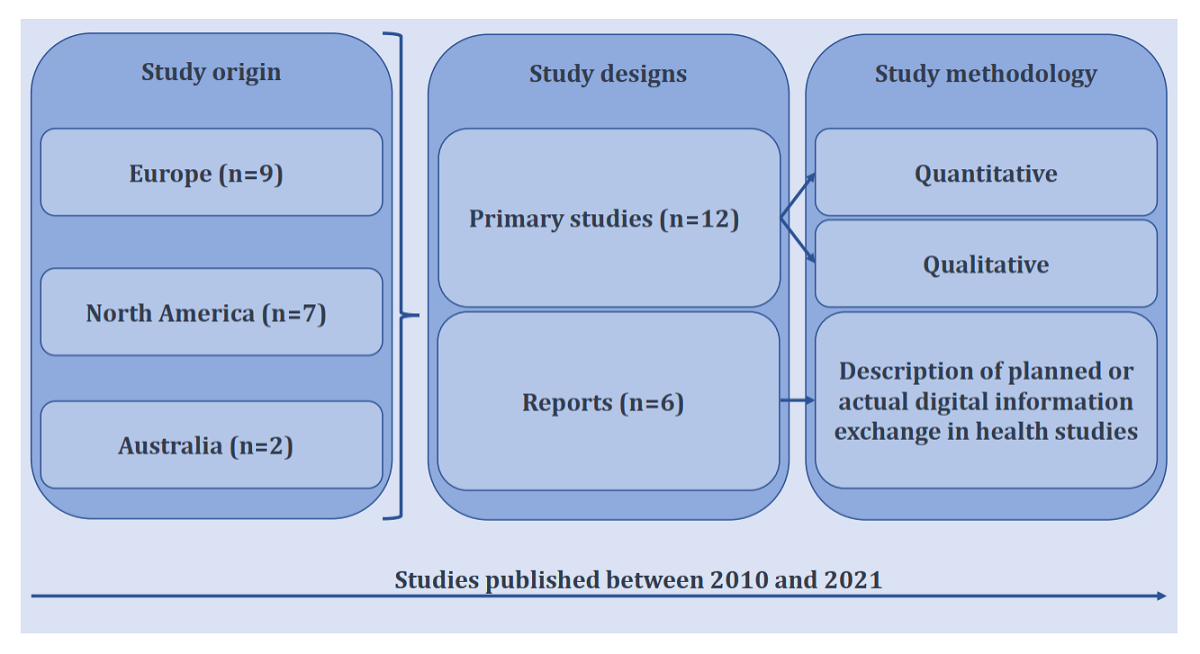
Characteristics of the 18 included studies.

All charted and processed data from the 18 studies are reported in Multimedia Appendix 4. The 18 studies were published between 2010 and 2021 (Figure 2). The studies originated from Europe (9/18, 50%), North America (7/18, 39%), and Australia (2/18, 11%). Funding source or a lack of funding was reported in 17 (94%) studies.

### Objective 1: Study Designs and Aims

The 18 studies included 12 (67%) primary studies [7,45,46,48,50,51,53,54,57,59-61] with quantitative or qualitative data and 6 (33%) reports [47,49,52,55,56,58], which described the actual or planned digital information exchange in health studies (Figure 2). Overall, 12 (67%) studies [45-47,49,54-61] aimed to evaluate the existing strategies for digital information exchange. The remaining 6 (33%) studies [7,48,50-53] assessed preferences for such information exchange.

### Objective 2: Population Characteristics

**Figure 3:**
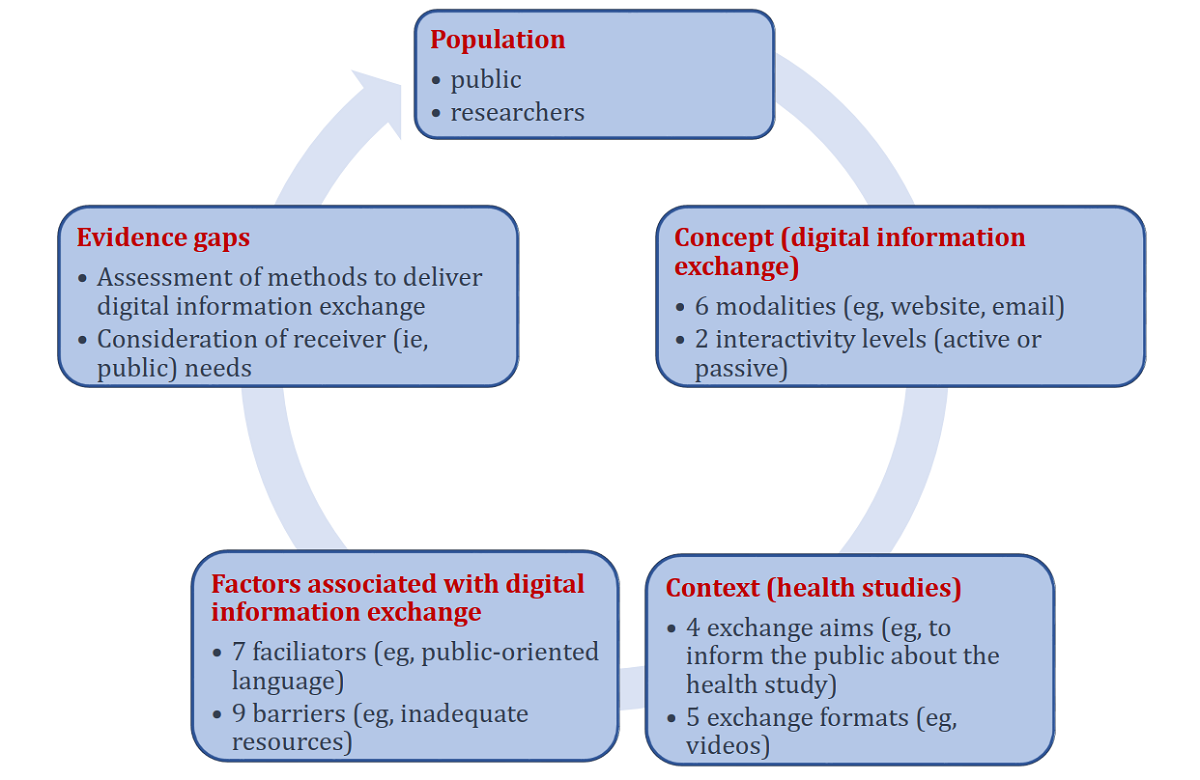
Characteristics of the 18 included studies based on objectives 2-6 in the scoping review.

The PCC characteristics of the included studies are summarized in Figure 3.

All 18 studies addressed digital information exchange for 1 to 2 (mean 1.3, SD 0.5) population types. The addressed populations included the public in 10 (56%) studies [45-47,50,51,54-57,59], the researchers in 2 (11%) [52,53], and both the public and researchers in 6 (33%) [7,48,49,58,60,61].

Among the 18 studies, 9 (50%) [7,46,47,49,54,56,58,59,61] focused on populations in Europe, 4 (22%) [7,51,53,55] focused on populations in North America, and 1 (6%) each focused on populations in Asia [50], Australia [48], or Africa [7]. Overall, 4 (22%) studies [45,52,57,60] did not state the region of the assessed population.

Moreover, among the 18 studies, 7 (39%) [48,50,51,59,60] addressed only adults or adolescents (ie, people aged 18 years or 16 years or older) and 3 (17%) [47,54,55] addressed people of any age (ie, children and adults). In 8 (44%) studies [45,46,49,52,56-58,61], the age of the addressed population was not mentioned.

Most of the 18 studies focused on clinical populations. Overall, 8 (44%) studies [7,47,49,51,53-56] addressed any diseases, 5 (28%) [46,48,57,58,60] addressed specific diseases (eg, rare diseases or chronic kidney disease), and 3 (17%) [45,59,61] addressed any cancer diagnoses. In 2 (11%) studies [50,52], the health focus was not mentioned.

### Objective 3: Concept (Digital Information Exchange Methods)

Among the 18 studies, 13 (72%) [45-47,49,51,54-61] described 1 to 6 (mean 1.6, SD 0.7) exchange modalities. All 13 studies described methods of digital information exchange using diverse terminology. According to this terminology, digital information exchange occurred through websites, emails, forums, platforms, social media, or portals (Table 3). Some of those studies (6/13, 46%) [47,54-56,58,61] described multiple modalities of digital information exchange.

**Table 3:** Digital information exchange methods.

Variable		Studies (N=18), n (%)	Study citation
**Modality**			
	Website	10 (56)	[45,47,49,54-59,61]
	Email	3 (17)	[47,51,54]
	Forum	3 (17)	[47,55,58]
	Platform	2 (11)	[46,60]
	Social media	2 (11)	[54,56]
	Portal	1 (6)	[61]
	Not mentioned	5 (28)	[7,48,50,52,53]
**Interactivity**			
	Active (eg, bidirectional communication via a website with a discussion forum)	4 (22)	[46,56,58,61]
	Passive (eg, 1-directional dissemination of information though a website)	3 (17)	[47,55,60]
	Any (active or passive)	2 (11)	[45,54]
Not mentioned		9 (50)	[7,48-53,57,59]

Interactivity in the method of digital information exchange was implicitly or explicitly addressed in 9 (50%) studies and not addressed in the remaining 9 (50%) studies. We classified the interactivity of digital information exchange as active, passive, or any (Table 3).

### Objective 4: Context (Content and Aim of Digital Information Exchange)

Among the 18 studies, 11 (61%) [45-47,49,55-57,59-61] mentioned exchange content (ie, data or information exchange regarding the health studies) and 7 (39%) [7,48,50-54] did not mention exchange content. Studies that addressed data exchange (3/11, 27%) [47,55,59] mentioned a digital exchange of individual and aggregated study data (1/3, 33%) [47] or only aggregated study data (2/3, 67%) [55,59].

Among the 11 studies [45-47,49,55-61] that addressed exchange content, there were 1 to 3 (mean 1.7, SD 0.7) exchange aims. The exchange aims focused on four themes: (1) to inform the public about the health study, (2) to recruit participants for the health study, (3) to link study participants with each other, and (4) to gather innovative research ideas from study participants (Table 4).

**Table 4:** Aim and format of digital information exchange.

Variable		Studies (N=18), n (%)	Study citation
**Exchange aim**			
	To inform the public about the health study	10 (56)	[45,47,49,55-61]
	To recruit participants for the health study	4 (22)	[49,58,60,61]
	To link study participants with each other	3 (17)	[46,47,61]
	To gather innovative research ideas from study participants	2 (11)	[46,58]
	Not mentioned	7 (39)	[7,48,50-54]
**Format**			
	Video	6 (33)	[46,47,55,56,58,61]
	Audio	3 (17)	[46,58,61]
	Picture	3 (17)	[46,47,58]
	Infographic	1 (6)	[55]
	Plain language summary	1 (6)	[58]
Not mentioned		12 (67)	[7,45,48-50,52-54,57,59,60]

Within these 11 studies that addressed exchange content, 6 (33%) [46,47,55,56,58,61] mentioned the format of exchange. Those 6 studies mentioned 1 to 4 (mean 2.3, SD 0.9) exchange formats that included videos, audio data, pictures, infographics, or plain language summaries (PLSs) (Table 4).

### Objective 5: Factors Associated With Digital Information Exchange

Among the 18 studies, we identified 7 facilitators of digital information exchange in 17 (94%) [7,46-61] and 9 barriers of digital information exchange in 16 (89%) [7,45,47,48,50-61]. There were 1 to 3 (mean 1.9, SD 0.8) facilitators and 1 to 5 (mean 2.3, SD 1.2) barriers of digital information exchange per study.

Among all 7 facilitators (Table 5; Multimedia Appendix 5), the most commonly identified facilitators in 6 of the 17 studies (35%) were: (1) consideration of any stakeholder perspectives and needs (by clarifying expectations and responsibilities), (2) use of modern or low-cost communication technologies, (3) use of public-oriented language, and (4) continuous communication of the health study process. Further facilitators identified in less than 6 of the 17 studies were: (1) appropriate dissemination of study results to participants, (2) appropriate amount of information content, and (3) interactive co-design of communication.

**Table 5:** Factors associated with digital information exchange.

Factors			Studies (N=18), n (%)		Study citation
**Facilitators of digital information exchange**					
	Consideration of any stakeholder perspectives and needs to clarify the expectations and responsibilities	6 (33)		[46,48,55,56,58,59]	
	Use of modern or low-cost communication technologies	6 (33)		[7,47,51,52,55,60]	
	Use of public-oriented language	6 (33)		[47,50,54,55,57,61]	
	Continuous communication of the health study process	6 (33)		[7,48,49,53,57,58]	
	Appropriate dissemination of study results to participants	4 (22)		[7,50,51,53]	
	Appropriate amount of information content	3 (17)		[47,54,61]	
	Interactive co-design of communication	2 (11)		[54,58]	
**Barriers of digital information exchange**					
	Information exchange not planned in study design or inadequate resources for planned information exchange	9 (50)		[7,48,50-55,61]	
	Too complex technical language for the public	6 (33)		[45,47,54,56,60,61]	
	Ethical concerns (eg, breach of anonymity if study participants are brought together)	5 (28)		[51-54,59]	
	Information exchange not wished from the public (eg, due to anticipated burden of potentially worrying research results, such as the detection of serious clinical symptoms)	4 (22)		[51,52,54,59]	
	Lack of interactive communication features	4 (22)		[45,54,58,61]	
	Low trust in digital health information	3 (17)		[58-60]	
	Poor health literacy among the public	2 (11)		[53,56]	
	Difficulties with finding or accessing digital health information	2 (11)		[57,60]	
	No possibility to contact the researchers	1 (6)		[45]	

Among all 9 barriers (Table 5; Multimedia Appendix 5), the most commonly identified barriers in 5 to 9 of the 16 studies were (1) information exchange not planned in study design or inadequate resources for planned information exchange, (2) too complex technical language for the public, and (3) ethical concerns. Other barriers identified in less than 5 of the 16 studies were (1) information exchange not wished from the public, (2) lack of interactive communication features, (3) low trust in digital health information, (4) poor health literacy among the public, (5) difficulties with finding or accessing digital health information, and (6) no possibility to contact the researchers.

### Objective 6: Evidence Gaps

Among the 18 studies, 9 (50%) [7,45-47,51-53,57,60] identified evidence gaps and provided suggestions for future research. Within these 9 studies, there were 1 to 2 (mean 1.2, SD 0.4) evidence gaps. These evidence gaps indicate that new studies are needed to address two themes: (1) assessment of methods to deliver and facilitate interactive digital information exchange and (2) consideration of receiver (ie, public) needs in the context of digital information exchange (Table 6).

**Table 6:** Evidence gaps and ideas for future research.

Evidence gaps	Studies (N=18), n (%)	Example statements from the included studies	Study citation
Assessment of methods to deliver and facilitate interactive digital information exchange	7 (39)	“[…], the quality of that information is highly variable, […], few sites offer interactive features. More research is needed to determine the best way of harnessing the power of the Internet—and its progeny, social media—to communicate high-yield cancer-related clinical trial information.” ([1], page 1631)	[45-47,51,53,57,60]
Consideration of receiver (ie, public) needs in the context of digital information exchange	3 (17)	“Studies […] identify participants’ specific goals with respect to using the information that they receive. Those studies could help researchers and funders gauge the extent to which they should focus on […] referring participants to specific resources, etc.” ([53], page 3)	[7,46,52]
No gaps identified	9 (50)	—^a^	[48-50,54-56,58,59,61]

^a^Not applicable.

## Discussion

### Principal Findings

The aim of this scoping review was to identify strategies for digital information exchange between the public and researchers in health studies. Our search identified only 18 peer-reviewed studies or reports that addressed various aspects of this topic. In general, very few details regarding such digital information exchange were reported. All studies described the actual or planned digital information exchange and focused mainly on receivers of health information, who were predominantly adult and clinical populations. The modality of digital information exchange was referred to using heterogeneous terminology, including websites, platforms, or portals. Exchange content included mostly health information and seldom study or individual patient data. Despite suggesting that digital information exchange should aim to inform, recruit, link, or gather innovative research ideas from participants in health studies, only half of the included studies addressed the issue of interactivity (ie, if digital information exchange is or should be active or passive). In addition, there were more barriers than facilitators associated with digital information exchange identified in the included studies. Thus, a key finding of this scoping review is the notable gap in evidence around standardized methods for digital information exchange in health studies. It is unclear how such digital information exchange should be designed and implemented across various platforms and audiences. Since digital platforms are increasingly used to share health research, evidence-based guidance on the best practices is needed to ensure that digital information exchange is effective, engaging, and accessible to a wide range of audiences, and contributes to long-term health benefits. This review identified several facilitators and barriers impacting digital information exchange that could be considered by researchers who plan such an exchange in their health studies. Overall, new studies are needed to assess the methods and public needs required to design, implement, and facilitate interactive digital information exchange.

### General Benefits of Digital Information Exchange in Health Studies

The past literature suggests that public participation and interest in clinical or medical trials has a positive impact on the visibility and influence of the trials [1,62] and that the aspect of participation has gained importance in recent decades, both scientifically and politically [8,63,64]. For example, digital information exchange between the public and researchers can enhance the inclusivity, accessibility, and acceptance of research among the public and ensure that research delivery aligns with their needs [62]. In addition, the COVID-19 pandemic has led to an increase in the development and use of digital portals with health data [33]. Digital information exchange methods like apps can facilitate communication between the public and health care providers by providing users access to health information, with consideration of challenges, such as privacy and security concerns, reliability, and accuracy of health information, through implementation to maximize their effectiveness [65].

### Content and Format of Digital Information Exchange in Health Studies

The studies included in this review suggest that digital information exchange should aim to inform, recruit, link, or gather innovative research ideas from participants in health studies. While most studies described the digital exchange of health information (eg, information on study content), only 3 studies addressed digital exchange of data [47,55,59]. Such data could include individual patient data [47] or aggregated study data [55,59]. In general, sharing of study data may be problematic due to ethical and legal reasons [66]. The investigation of study participant preferences for receiving research results [51] showed a spectrum of positive and negative responses, including anxiety, anger, guilt, relief, and pleasure, associated with receiving research results. Despite some negative responses, a median of 90% of participants reported the need to receive study results [51]. However, generalizing the results of studies on participant reactions and preferences regarding receiving research results is challenging due to relatively small sample sizes in such studies and a focus on specific and sensitive issues relevant for the targeted populations. Thus, different target populations may have potentially different needs for obtaining any study results or individual data.

Several formats of digital information exchange are available due to technological advancements, including not only text-based information but also other formats identified in this review, such as videos, blogs, and graphics. Given the effectiveness of PLSs based on comprehension, understanding, and enjoyment over graphical abstracts or published abstracts [67] and the availability of various PLS templates for several decades [68], it is surprising that this form of digital information exchange was mentioned in only 1 study in this review [58]. We have shown that studies with health content, such as Cochrane reviews that have mandatory PLSs according to Cochrane guidelines, are frequently mentioned online via channels accessible by the public (eg, social media, blogs, news, Wikipedia, and others) [69]. Although it is unclear how the public uses such information, exposure to information on health studies written in a nontechnical language could positively affect digital information exchange in terms of informing the public about health research. Health studies planning the digital information exchange of health data should consider the needs of different groups of relevant stakeholders. For example, target group–specific language should be used, and a lack of such language was frequently identified as a barrier to digital information exchange in this scoping review. This addresses both the lack of consideration mentioned above and the added value of this form of communication, which has been confirmed in previous studies [57,67].

According to the findings of this review, videos were the most used digital information exchange method. As suggested by others [67], if the research findings are of public relevance, researchers might consider investing time and money into creating a video of their results, as videos make viewers feel confident and positive about the presented research. However, other formats and methods, such as communication via interactive apps with gamification [70], could also be used in digital information exchange, enabling direct feedback options.

### Facilitators and Barriers of Digital Information Exchange in Health Studies

The results of this scoping review showed that websites, forums, and portals are the main methods of digital information exchange for connecting with the desired audience online. Despite the benefits of interaction as a form of participation (by or for the public) [71], only half of the identified studies provided information on the possibility of active or passive interaction (Table 3). Thus, despite the social relevance and the potential benefits, digital interaction between information providers (ie, researchers) and receivers (ie, the public) was neglected in the identified studies. Another review [72], which included 28 studies on patient participation, indicated that an average of 90% of participants were interested in their own or general study results. Another study also showed that individuals who requested study results did not receive feedback on these requests [51]. There could be several explanations for this finding as evident in the list of facilitators and barriers associated with digital information exchange identified in this review (Table 5). The main barrier to dissemination was that communication was not planned in the initial study design. Research suggests that even when community-based participatory research is conducted with public involvement as a central element, no more information is disseminated than is actually published in the form of a scientific publication [73]. One reason for this could be that study participants explicitly express no interest in receiving their own study results. This could be due to anticipated burden of potentially worrying research results, such as the detection of serious clinical symptoms [74].

Other key aspects that should be considered to improve the communication of study results and the communication of health information are adequate interaction with the information provided and an appropriate volume of such information. The latter is also a barrier to the dissemination of information and can be related to the poor health literacy among the public. Information can be burdensome when it seems too complex and not tangible [75]. The filtering function could be used to counteract potential information overload [76]. This approach suggests that interactivity rather than a passive provision of health information is necessary. Incorporating interactivity when sharing research studies with the public can increase engagement, improve understanding, and make complex information more accessible [77]. Examples of interactive communication methods are interactive graphs and maps that show data over time (eg, COVID-19 spread) to help users explore trends [78], interactive quizzes [79], and serious games related to study content that can improve health literacy or online forums where people can discuss the research findings, give feedback, and see responses [80].

This scoping review also identified several facilitators that can strengthen communication between researchers and the public as well as increase trust in the information presented online (on websites or portals). In particular, the trustworthiness of information is a relevant aspect to be addressed in future studies [81]. To improve trust in health information online, such information could be discussed with health professionals who continue to be important reference persons [82].

### Evidence Gaps

Evidence gaps in the included studies suggest that appropriate methods required to deliver and facilitate interactive digital information exchange need to be identified in future research. Furthermore, the preferences and needs of the receiver (ie, public) to facilitate such digital information exchange should be investigated. Interestingly, the consideration of the preferences and needs of the target populations (ie, the public being the health information receivers) was mentioned as a requirement for future studies already several years ago [7,46,52] and was the most commonly reported facilitator of digital information exchange in this review (Table 4).

### Strengths and Limitations

The main strength of this review is that it addresses a highly relevant topic on how information generated in health studies can be exchanged between the public and researchers using digital methods. In particular, we identified a detailed list of facilitators and barriers associated with digital information exchange that could be considered in health studies. The literature search was supervised and performed by an experienced information specialist, and a standardized methodological approach was used to select studies and extract, process, and synthesize the data.

The main limitation of this review is the difficulty in finding relevant studies in bibliographic databases. Identifying the most suitable terminology for our objectives was challenging because some of the less specific search terms, such as public and health studies, frequently appear in diverse contexts within the health literature. Despite numerous iterations, our search syntax identified only 18 studies published up to 2021 that met the inclusion criteria. It cannot be ruled out that we missed other published studies that did not include our search terms in their titles, abstracts, or other searched fields. However, the peer-reviewed literature on this topic might be truly limited, as it is possible that researchers do not report how they share research results with the public in their studies even if they do so. Thus, we would like to encourage researchers working on health studies to comment on and share their experiences regarding digital information exchange from such studies in academic articles. Other research designs, such as comprehensive assessments of existing online data sharing databases and portals and qualitative interviews with health information providers and receivers, could be used to further investigate digital information exchange in health studies as the field evolves.

Furthermore, there was relatively little information available on the details of digital information exchange in the included studies. Despite this, we identified various factors that may help other researchers to facilitate digital information exchange and reduce the barriers in such an exchange. Although our list of such facilitators and barriers is extensive, further factors may exist that need to be identified in future research. Finally, probably due to the fact that the included studies were somewhat dated (ie, published up to 2021), we could not identify any studies discussing more recent methods of digital information exchange, such as study-related communication via apps or the use of artificial intelligence in supporting such communication. One example of a relevant app is PIA (Prospective Monitoring and Management App) developed for monitoring, managing observational epidemiological studies, and systematically collecting user feedback [70].

### Conclusions

This scoping review describes how digital information exchange occurs between the public and researchers in health studies. Our search strategy identified only 18 studies on this topic that were published mostly in Western countries (Europe and North America) between 2010 and 2021. In these studies, there was little focus on interactivity in digital information exchange, and more barriers than facilitators of such an exchange were identified. The facilitators and barriers associated with digital information exchange offer crucial insights for gaining a deeper understanding of how effective digital information exchange can be designed and implemented. Given the evidence gaps identified in the included studies, further research is needed to explore how to improve digital information exchange between the public and researchers. Future studies should investigate interactive digital methods and receiver preferences and needs required for such an exchange.

## Data Availability

The datasets generated and analyzed during this study are reported in the article and multimedia appendices.

## Appendix

Multimedia Appendix 1 PRISMA-ScR (Preferred Reporting Items for Systematic Reviews and Meta-Analyses Extension for Scoping Reviews) checklist.

Multimedia Appendix 2 Search strategy.

Multimedia Appendix 3 List of included and excluded studies.

Multimedia Appendix 4 Data file.

Multimedia Appendix 5 Facilitators and barriers of digital information exchange with examples.

## Abbreviations

EHR: electronic health record
NFDI4Health: National Research Data Infrastructure for Personal Health Data
PCC: Population, Concept, and Context
PLS: plain language summary
PRISMA-ScR: Preferred Reporting Items for Systematic Reviews and Meta-Analyses Extension for Scoping Reviews
