# Supplementary material for: Digital Information Exchange Between the Public and Researchers in Health Studies: Scoping Review
Source: J Med Internet Res. 2025 Jan 28;27:e63373. doi: 10.2196/63373 (PMC11815310; doi:10.2196/63373)
Supplement: Multimedia Appendix 3 [file jmir_v27i1e63373_app3.docx]

**Multimedia Appendix 3.** List of included and excluded studies.

| **Study status** | **Studies, n** | **Citation** |
| --- | --- | --- |
| **Identified studies** | **4072** |  |
| Studies excluded title/abstract | 4024 |  |
| **Studies screened full-text** | **48** |  |
| **Included studies after full-text search** | **18** | **[1-18]** |
| From bibliographic databases | 13 | **[1, 2, 6-10, 12-15, 17, 18]** |
| From manual searches | 5 | **[3-5, 11, 16]** |
| **Excluded studies after full-text screening** | **30** |  |
| Exclusion 3: Other context: 1) no focus on research studies | 22 | [19-40] |
| Exclusion 3: Other context: 2) focus on other specific topics in the health context, including clinical treatment, development of health systems, health frameworks, digital health technologies, clinical guidelines, procedures, or tools; marketing and sales of health products; ethical and legal issues in consent and data sharing; teaching, training, and health education for healthcare professionals; recruitment of participants for research or clinical treatment; health literacy; online support groups; quality of health information; focus on personal data, e.g., electronic health records or genome data) | 8 | [41-48] |

## References

1. Abel GA, Cronin AM, Earles K, Gray SW. Accessibility and Quality of Online Cancer-Related Clinical Trial Information for Naive Searchers. Cancer Epidemiology, Biomarkers & Prevention. 2015 Oct;24(10):1629-31. doi: <https://dx.doi.org/10.1158/1055-9965.EPI-15-0274>.

2. Bullinger AC, Rass M, Adamczyk S, Moeslein KM, Sohn S. Open innovation in health care: analysis of an open health platform. 2012 May;1(2-3):165-75. doi: 10.1016/j.healthpol.2012.02.009.

3. Dietrich J, Alivojvodic J, Seliverstov I, Metcalf M, Jakee K. Improving Information Exchange with Clinical Trial Participants: A Proposal for Industry. Ther Innov Regul Sci. 2017 2017/09//;51(5):542-50. doi: 10.1177/2168479017725109.

4. Exley K, Cano N, Aerts D, Biot P, Casteleyn L, Kolossa-Gehring M, et al. Communication in a Human biomonitoring study: Focus group work, public engagement and lessons learnt in 17 European countries. Environmental Research. 2015 2015/08//;141:31-41. doi: 10.1016/j.envres.2014.12.003.

5. Gutman T, Tong A, Howell M, Dansie K, Hawley CM, Craig JC, et al. Principles and strategies for involving patients in research in chronic kidney disease: report from national workshops. Nephrology Dialysis Transplantation. 2020 2020/09/01/;35(9):1585-94. doi: 10.1093/ndt/gfz076.

6. Hägglund M, Scott Duncan T, Kai-Larsen K, Hedlin G, Krakau I. IntegrIT - Towards Utilizing the Swedish National Health Information Exchange Platform for Clinical Research. Stud Health Technol Inform. 2017 2017;235:146-50.

7. Keys JR, Monk JA, Woolley KL. Sharing Results with Clinical Trial Participants: Insights from an Online Survey of Chinese Consumers. Chinese Medical Journal. 2016 Apr 20;129(8):1007-8. doi: <https://dx.doi.org/10.4103/0366-6999.179787>.

8. Long CR, Purvis RS, Flood-Grady E, Kimminau KS, Rhyne RL, Burge MR, et al. Health researchers’ experiences, perceptions and barriers related to sharing study results with participants. Health Res Policy Sys. 2019 2019/12//;17(1):25. doi: 10.1186/s12961-019-0422-5.

9. Long CR, Stewart MK, Cunningham TV, Warmack TS, McElfish PA. Health research participants’ preferences for receiving research results. Clinical Trials. 2016 2016/12//;13(6):582-91. doi: 10.1177/1740774516665598.

10. Long CR, Stewart MK, McElfish PA. Health research participants are not receiving research results: a collaborative solution is needed. Trials. 2017 2017/12//;18(1):449. doi: 10.1186/s13063-017-2200-4.

11. Lucas PJ, Allnock D, Jessiman T. How are European birth-cohort studies engaging and consulting with young cohort members? BMC Med Res Methodol. 2013 2013/12//;13(1):56. doi: 10.1186/1471-2288-13-56.

12. Monnard K, Benjamins MR, Hirschtick JL, Castro M, Roesch PT. Co-Creation of Knowledge: A Community-Based Approach to Multilevel Dissemination of Health Information. Health Promotion Practice. 2021 2021/03//;22(2):215-23. doi: 10.1177/1524839919865228.

13. Mosconi P, Antes G, Barbareschi G, Burls A, Demotes-Mainard J, Chalmers I, et al. A European multi-language initiative to make the general population aware of independent clinical research: the European Communication on Research Awareness Need project. Trials. 2016 2016/12//;17(1):19. doi: 10.1186/s13063-015-1146-7.

14. Penlington M, Silverman H, Vasudevan A, Pavithran P. Plain Language Summaries of Clinical Trial Results: A Preliminary Study to Assess Availability of Easy-to-Understand Summaries and Approaches to Improving Public Engagement. Pharmaceutical Medicine. 2020 12;34(6):401-6. doi: <https://dx.doi.org/10.1007/s40290-020-00359-4>.

15. Rubinelli S, Collm A, Glässel A, Diesner F, Kinast J, Stucki G, et al. Designing interactivity on consumer health websites: PARAFORUM for spinal cord injury. Patient Education & Counseling. 2013;93(3):459-63. doi: 10.1016/j.pec.2013.09.015.

16. Sarradon-Eck A, Sakoyan J, Desclaux A, Mancini J, Genre D, Julian-Reynier C. "They should take time": disclosure of clinical trial results as part of a social relationship. Soc Sci Med. 2012 Sep;75(5):873-82. doi: 10.1016/j.socscimed.2012.04.022.

17. Suart CE, Graham KJ, Suart TN, Truant R. Development of a knowledge translation platform for ataxia: Impact on readers and volunteer contributors. PLOS ONE. 2020 2020/09/01/;15(9):e0238512. doi: 10.1371/journal.pone.0238512.

18. Wallwiener M, Wallwiener CW, Brucker SY, Hartkopf AD, Fehm TN, Kansy JK. The Brustkrebs-Studien.de website for breast cancer patients: User acceptance of a German internet portal offering information on the disease and treatment options, and a clinical trials matching service. 2010 Dec 02;1:663.

19. Alba-Ruiz R, Bermúdez-Tamayo C, Pernett JJ, Garcia-Gutierrez JF, Cózar-Olmo JM, Valero-Aguilera B. Adapting the Content of Cancer Web Sites to the Information Needs of Patients: Reliability and Readability. Telemedicine and E-Health. 2013 Dec;19(12):956-66. doi: 10.1089/tmj.2013.0050.

20. Allison KR, Patterson P, Ussher JM, McDonald FE, Perz J. Evaluating Maybe Later Baby, a fertility information resource for adolescents and young adults diagnosed with cancer: A randomized, controlled pilot study. Journal of Adolescent and Young Adult Oncology. 2023 Feb;12(1):101-9. doi: <https://dx.doi.org/10.1089/jayao.2021.0206>.

21. Amr A, Hinderer M, Griebel L, Deuber D, Egger C, Sedaghat-Hamedani F, et al. Controlling my genome with my smartphone: first clinical experiences of the PROMISE system. Clinical Research in Cardiology. 2022 Jun;111(6):638-50. doi: <https://dx.doi.org/10.1007/s00392-021-01942-8>.

22. Babac A, Litzkendorf S, Schmidt K, Pauer F, Damm K, Frank M, et al. Shaping an Effective Health Information Website on Rare Diseases Using a Group Decision-Making Tool: Inclusion of the Perspectives of Patients, Their Family Members, and Physicians. Interactive Journal of Medical Research. 2017 Nov 20;6(2):e23. doi: <https://dx.doi.org/10.2196/ijmr.7352>.

23. Barriguete-Meléndez JA, Hercberg S, Galán P, Parodi A, Baulieux J, Abdo M, et al. NutriNet-Salud Mexico. Prospective study online: 2018-2028. Relationship between nutrition and health and determinants of dietary habits and nutritional status. Cirugia Y Cirujanos. 2018 Jan-Feb;86(1):4-14. doi: 10.24875/cirue.M18000002.

24. Briggs AM, March L, van den Haak R, Hay N, Henderson L, Murphy B, et al. Stakeholder satisfaction with the Australian Rheumatology Association Database (ARAD). The Patient: Patient-Centered Outcomes Research. 2009 Jan;2(1):61-8. doi: <https://dx.doi.org/10.2165/01312067-200902010-00007>.

25. Burgun A, Oksen DV, Kuchinke W, Prokosch HU, Ganslandt T, Buchan I, et al. Proposal for a European Public Health Research Infrastructure for Sharing of health and Medical administrative data (PHRIMA). Studies in Health Technology & Informatics. 2015;216:1005.

26. Correa DJ, Milano L, Kwon CS, Jette N, Dlugos D, Harte-Hargrove L, et al. Quantitative readability analysis of websites providing information on traumatic brain injury and epilepsy: A need for clear communication. Epilepsia. 2020 Mar;61(3):528-38. doi: 10.1111/epi.16446.

27. de Hosson LD, Bouma G, Stelwagen J, van Essen H, de Bock GH, de Groot DJA, et al. Web-based personalised information and support for patients with a neuroendocrine tumour: randomised controlled trial. Orphanet Journal Of Rare Diseases. 2019 02 28;14(1):60. doi: <https://dx.doi.org/10.1186/s13023-019-1035-3>.

28. Engholm G, Ferlay J, Christensen N, Bray F, Gjerstorff ML, Klint A, et al. NORDCAN--a Nordic tool for cancer information, planning, quality control and research. 2010 Jun;1(5):725-36.

29. Fedorov A, Longabaugh W, Pot D, Clunie D, Pieper S, Lewis R, et al. NCI Imaging Data Commons. International Journal of Radiation Oncology, Biology, Physics. 2021;111(3):e101-e. doi: 10.1016/j.ijrobp.2021.07.495.

30. Fleisher L, Kenny C, Rusten C, Koren D, Landau Z. Right Information, Right Patient, Right Time: Utilizing the MyCareCompass Platform to Deliver Patient Education in the Oncology Setting. Journal of Cancer Education. 2023 Oct;38(5):1420-8. doi: 10.1007/s13187-023-02350-4.

31. Haase J, Wagner TOF, Storf H. [se-atlas - the health service information platform for people with rare diseases : Supporting research on medical care institutions and support groups]. Bundesgesundheitsblatt, Gesundheitsforschung, Gesundheitsschutz. 2017 May;60(5):503-9. doi: <https://dx.doi.org/10.1007/s00103-017-2529-6>.

32. Khurshid A, Nauman E, Carton T, Horswell R. Louisiana Clinical Data Research Network: establishing an infrastructure for efficient conduct of clinical research. Journal of the American Medical Informatics Association. 2014 Jul-Aug;21(4):612-4. doi: <https://dx.doi.org/10.1136/amiajnl-2014-002740>.

33. Lelong R, Soualmia LF, Grosjean J, Taalba M, Darmoni SJ. Building a Semantic Health Data Warehouse in the Context of Clinical Trials: Development and Usability Study. Jmir Medical Informatics. 2019 Oct-Dec;7(4):153-69. doi: 10.2196/13917.

34. Ma C, Sridharan M, Al-Sayegh H, Li A, Guo D, Auclair M, et al. Building a Harmonized Datamart by Integrating Cross-Institutional Systems of Clinical, Outcome, and Genomic Data: The Pediatric Patient Informatics Platform (PPIP). JCO Clinical Cancer Informatics. 2021 02;5:202-15. doi: <https://dx.doi.org/10.1200/CCI.20.00083>.

35. Magal Royo T, Cortell Sivera A, García Laborda A, Prefasi Gomar S, Garde Calvo F. A Web platform of the Valencia local government for the promotion of contents on mental health. Metas de Enfermería. 2010;13(1):16-20.

36. Rommel K, Nyoungui E, Dierking A, Langenbeck U, Schmidtke J, Stuhrmann M. Orphanet - the European portal for rare diseases. A communication tool. Medizinische Genetik. 2010 Jun;22(2):213-+. doi: 10.1007/s11825-010-0219-y.

37. Seckin G. Health information on the web and consumers' perspectives on health professionals' responses to information exchange. Medicine 20. 2014 Jul-Dec;3(2):e4. doi: <https://dx.doi.org/10.2196/med20.3213>.

38. Visser M, Kotze M, van Rensburg MJ. An mHealth HIV prevention programme for youth: lessons learned from the iloveLife.mobi programme in South Africa. AIDS Care. 2020;32:148-54. doi: 10.1080/09540121.2020.1742866.

39. Wang T, Lund B, Dow M. Improving Health Information for Rare Disease Patients and Caregivers: A Survey of Preferences for Health Information Seeking Channels and Formats. Journal of Hospital Librarianship. 2023;23(2):83-96. doi: 10.1080/15323269.2023.2194808.

40. Williams P, Nicholas D, Huntington P, McLean F. Surfing for health: user evaluation of a health information website. Part two: fieldwork. Health Information & Libraries Journal. 2002;19(4):214-25.

41. Ali J, Califf R, Sugarman J. Anticipated Ethics and Regulatory Challenges in PCORnet: The National Patient-Centered Clinical Research Network. Accountability in Research. 2016;23(2):79-96. doi: <https://dx.doi.org/10.1080/08989621.2015.1023951>.

42. Atkinson NL, Massett HA, Mylks C, McCormack LA, Kish-Doto J, Hesse BW, et al. Assessing the impact of user-centered research on a clinical trial eHealth tool via counterbalanced research design. Journal of the American Medical Informatics Association. 2011 2011/01//;18(1):24-31. doi: 10.1136/jamia.2010.006122.

43. Forrest CB, McTigue KM, Hernandez AF, Cohen LW, Cruz H, Haynes K, et al. PCORnet R 2020: current state, accomplishments, and future directions. Journal of Clinical Epidemiology. 2021 01;129:60-7. doi: <https://dx.doi.org/10.1016/j.jclinepi.2020.09.036>.

44. George S, Moran E, Duran N, Jenders RA. Using animation as an information tool to advance health research literacy among minority participants. AMIA Annu Symp Proc. 2013 2013;2013:475-84.

45. Haga SB, Friedman B, Richard G. Considering the Benefits and Risks of Research Participants' Access to Sequence Data. Genetic Testing and Molecular Biomarkers. 2017 2017/12//;21(12):717-21. doi: 10.1089/gtmb.2017.0143.

46. Mai PL, Sand SR, Saha N, Oberti M, Dolafi T, DiGianni L, et al. Li-Fraumeni Exploration Consortium Data Coordinating Center: Building an Interactive Web-Based Resource for Collaborative International Cancer Epidemiology Research for a Rare Condition. Cancer Epidemiology, Biomarkers & Prevention. 2020 05;29(5):927-35. doi: <https://dx.doi.org/10.1158/1055-9965.EPI-19-1113>.

47. Öfelein M, Reichold M, Maier C, Prokosch H-U. Designing a Framework of Components to Support Patient Engagement in Research. Stud Health Technol Inform. 2019 2019/09/03/;267:20-7. doi: 10.3233/SHTI190800.

48. Statham EE, White SA, Sonwane B, Bierer BE. Primed to comply: Individual participant data sharing statements on ClinicalTrials.gov. PLoS ONE [Electronic Resource]. 2020;15(2):e0226143. doi: <https://dx.doi.org/10.1371/journal.pone.0226143>.
