# Supplementary material for: Digital Information Exchange Between the Public and Researchers in Health Studies: Scoping Review
Source: J Med Internet Res. 2025 Jan 28;27:e63373. doi: 10.2196/63373 (PMC11815310; doi:10.2196/63373)
Supplement: Multimedia Appendix 5 [file jmir_v27i1e63373_app5.docx]

**Multimedia Appendix 5.** Facilitators and barriers of digital information exchange with examples.

| **Factors** | **n studies** | **% of 18** | **Example statements from study authors (citation)** | **References** |
| --- | --- | --- | --- | --- |
| **Facilitators of digital information exchange** | | | | |
| 1. Consideration of any stakeholder perspectives and needs (by clarifying expectations and responsibilities) | 6 | 33.3% | “Engaging patients in research could be improved if researchers facilitated the development of a community for research involvement and ‘took research to the patients’ on their own terms in a convenient, informal and unintimidating environment.” [1]  “’*Clarifying expectations and responsibilities*’: […] They suggested to clarify the patient’s role, expected commitments and potential impact of their involvement to encourage patient research partners.” [1] | [1–6] |
| 1. Use of modern or low-cost communication technologies | 6 | 33.3% | “Current volunteers reported a key strength of SCAsource was the opportunity to practice knowledge translation. This opportunity […] giving them a supportive environment with constructive feedback to improve their lay summary writing. This is further reflected by the request for more extensive knowledge translation training for volunteers.” [7] | [3,7–11] |
| 1. Use of public-oriented language | 6 | 33.3% | “Understandable, easy-to-read summaries of clinical research have been recognized as providing greater transparency to those interested in learning about clinical trial results.” [12] | [3,9,12–15] |
| 1. Continuous communication of health study process | 6 | 33.3% | “The national Swedish HIE platform has the potential to provide […] useful tools  for clinicians […] and patients […] but also to enable a more seamless connection between patients/citizens as study participants, health care professionals and everyday clinical work and clinical researchers in both academia and industry.” [16]  “[…], the information preferences of all respondents remained largely similar and in the same order of preference, with minor exceptions.   - By email - From the clinical study staff/study doctor - Online, with a secure username and password to access the information Live phone call (speak to a person) - By postal mail - From my usual / primary health care professional / doctor - Through an app on my smartphone - By text message   Automated phone call” [8 ff.] | [1,5,8,12,16,17] |
| 1. Appropriate dissemination of study results to participants | 4 | 22.2% | “Sharing clinical trial results with participants has many benefits, […] demonstrating respect and appreciation for participation; facilitating communication between clinicians and participants; and increasing patient satisfaction with trial participation, […].” [13] | [8,10,13,17] |
| 1. Appropriate amount of information content | 3 | 16.7% | “Lesson learnt: Do not overload potential participants with too much information. Provide initial information that is friendly […], simple and short. If people are interested […], then provide them with more detailed information”. [9 ff.] | [9,14,15] |
| 1. Interactive co-design of communication | 2 | 11.1% | “These last two dimensions aim to promote the growth of a collaborative online community where the expertise […] is identified and translated to enhance new ideas for research and organizations’ practice. Through interactions regarding specific problems and challenges, users can co-design solutions” [5] | [5,14] |
| **Barriers of digital information exchange** | | | | |
| 1. Information exchange not planned in study design or inadequate resources for planned information exchange | 9 | 50% | “For those participants who may intend to act, it also unclear how, or whether, to present specific information to contextualize the results, including limitations on the generalizability of results, the need for replication, or comparative effectiveness with other treatments or interventions. If results are to be communicated effectively, participants’ intentions—which likely vary according to participant population, research design, and context—must be better understood.” [11]  “Partnering with patients in research requires respect and recognition of their unique, diverse and complementary experiential expertise. Multiple and flexible approaches are necessary to consider the patient circumstances, […] and existing burdens of their illness and treatment. Harnessing relationships with trusted clinicians and community organizations may help[…], while innovative infrastructure […] can be further explored. Robust tools and frameworks to evaluate patient involvement in research are also needed.” [1 ff.]  “However, the notion that patients need to be ‘representative’ to be able to make a meaningful contribution to research was challenged. It was argued that the intrinsic value […] experience that patients bring as research partners can serve to ground research in real-world applications, resulting in research that is relevant and important to all stakeholders […].” [1] | [1,3,8,10,11,13–15,17] |
| 1. Too complex technical language for the public | 6 | 33.3% | “However, complex trial descriptions and eligibility criteria, the extensive use of medical terminology and expecting patients to determine preferences early on in the registration process act as strong barriers to trial participation." [15] | [4,7,9,14,15,18] |
| 1. Ethical concerns | 5 | 27.8% | “In addition a number of ethical considerations were raised: […]; there was also a concern that any activity that might bring together study members would breach anonymity; and finally there were felt to be ethical constraints in working with the children independent of their parents.” [14] | [6,10,11,14,17] |
| 1. Information exchange not wished from the public | 4 | 22.2% | “Their refusal or omission to consult the electronic document may have been due to their distancing of their cancer episode. They expressed the need to “move on” and “to forget” the illness, “to let it go”. This coping strategy alleviates the emotional load […] and decreases their fear of relapse.” [6] | [6,10,11,14] |
| 1. Lack of interactive communication features | 4 | 22.2% | “On the other hand, the quality of that information is highly variable, and for some cancers, few sites offer interactive features. […]. Finally, given that a significant portion of interactive sites found through naive searches ultimately link to NCI's databases, the quality of the information preceding that database link may merit careful review” [18]  “In producing these websites, health organizations invest significant resources to provide health information in the traditional one-way […] model of communication. However, as shown in the past few years one-way communication as a channel for influencing health behavior has limitations” [5 ff.] | [5,14,15,18] |
| 1. Low trust in digital health information | 3 | 16.7% | “[…], the present trial participants’ attitudes towards computer technology indicate that they had greater trust in information given by doctors, […] because […] several websites can provide contradictory information” [6] | [5–7] |
| 1. Poor health literacy in the public | 2 | 11.1% | “Researchers are open to sharing results with participants, but they perceive significant barriers to sharing; […]. Among respondents who expressed reluctance about sharing results with participants, the most common concerns related to participants’ health literacy or ability to comprehend research findings. To address these concerns, academic research institutions with CTSAs should take a leading role.” [17] | [4,17] |
| 1. Difficulties with finding or accessing digital health information | 2 | 11% | “In the absence of comprehensive published accounts, we can only guess how the public will search for information. Even then, search hits can vary among individuals for a given query because ever-evolving search engine algorithms personalize user experience. However, this is unlikely to be beneficial if the material cannot be easily found in the first place” [12]  “There are several barriers facing laypersons trying to access research information online. Often laypersons run into paywalls when trying to access primary research […].” [7] | [7,12] |
| 1. No possibility to contact the researchers | 1 | 5.6% | “In addition, given that patients desire to be well informed about the potential financial aspects of investigators and trial sponsors, while it was reassuring that sponsors were always listed, we were disappointed to find that the principal investigator was named on fewer than 2 out of 3 websites. [18] | [18] |

## References

1. Gutman T, Tong A, Howell M, Dansie K, Hawley CM, Craig JC, Jesudason S, Chapman JR, Johnson DW, Murphy L, Reidlinger D, Crowe S, Duncanson E, Muthuramalingam S, Scholes-Robertson N, Williamson A, McDonald S. Principles and strategies for involving patients in research in chronic kidney disease: report from national workshops. Nephrol Dial Transplant 2020 Sep 1;35(9):1585–1594. doi: 10.1093/ndt/gfz076

2. Bullinger AC, Rass M, Adamczyk S, Moeslein KM, Sohn S. Open innovation in health care: analysis of an open health platform. 2012 May;1(2–3):165–75. doi: 10.1016/j.healthpol.2012.02.009

3. Monnard K, Benjamins MR, Hirschtick JL, Castro M, Roesch PT. Co-Creation of Knowledge: A Community-Based Approach to Multilevel Dissemination of Health Information. Health Promot Pract 2021;22(2):215–223. doi: 10.1177/1524839919865228

4. Mosconi P, Antes G, Barbareschi G, Burls A, Demotes-Mainard J, Chalmers I, Colombo C, Garattini S, Gluud C, Gyte G, McLlwain C, Penfold M, Post N, Satolli R, Valetto MR, West B, Wolff S. A European multi-language initiative to make the general population aware of independent clinical research: the European Communication on Research Awareness Need project. Trials 2016;17(1):19. doi: 10.1186/s13063-015-1146-7

5. Rubinelli S, Collm A, Glässel A, Diesner F, Kinast J, Stucki G, Brach M. Designing interactivity on consumer health websites: PARAFORUM for spinal cord injury. Patient Educ Couns 2013;93(3):459–463. doi: 10.1016/j.pec.2013.09.015

6. Sarradon-Eck A, Sakoyan J, Desclaux A, Mancini J, Genre D, Julian-Reynier C. “They should take time”: Disclosure of clinical trial results as part of a social relationship. Soc Sci Med 2012 Sep;75(5):873–882. doi: 10.1016/j.socscimed.2012.04.022

7. Suart CE, Graham KJ, Suart TN, Truant R. Development of a knowledge translation platform for ataxia: Impact on readers and volunteer contributors. Harris KM, editor. PLOS ONE 2020 Sep 1;15(9):e0238512. doi: 10.1371/journal.pone.0238512

8. Dietrich J, Alivojvodic J, Seliverstov I, Metcalf M, Jakee K. Improving Information Exchange with Clinical Trial Participants: A Proposal for Industry. Ther Innov Regul Sci 2017;51(5):542–550. doi: 10.1177/2168479017725109

9. Exley K, Cano N, Aerts D, Biot P, Casteleyn L, Kolossa-Gehring M, Schwedler G, Castaño A, Angerer J, Koch HM, Esteban M, Schoeters G, Den Hond E, Horvat M, Bloemen L, Knudsen LE, Joas R, Joas A, Dewolf M-C, Van de Mieroop E, Katsonouri A, Hadjipanayis A, Cerna M, Krskova A, Becker K, Fiddicke U, Seiwert M, Mørck TA, Rudnai P, Kozepesy S, Cullen E, Kellegher A, Gutleb AC, Fischer ME, Ligocka D, Kamińska J, Namorado S, Fátima Reis M, Lupsa I-R, Gurzau AE, Halzlova K, Jajcaj M, Mazej D, Tratnik JS, Huetos O, López A, Berglund M, Larsson K, Sepai O. Communication in a Human biomonitoring study: Focus group work, public engagement and lessons learnt in 17 European countries. Environ Res 2015;141:31–41. doi: 10.1016/j.envres.2014.12.003

10. Long CR, Stewart MK, Cunningham TV, Warmack TS, McElfish PA. Health research participants’ preferences for receiving research results. Clin Trials 2016;13(6):582–591. doi: 10.1177/1740774516665598

11. Long CR, Stewart MK, McElfish PA. Health research participants are not receiving research results: a collaborative solution is needed. Trials 2017;18(1):449. doi: 10.1186/s13063-017-2200-4

12. Penlington M, Silverman H, Vasudevan A, Pavithran P. Plain Language Summaries of Clinical Trial Results: A Preliminary Study to Assess Availability of Easy-to-Understand Summaries and Approaches to Improving Public Engagement. Pharm Med 2020;34(6):401–406. doi: https://dx.doi.org/10.1007/s40290-020-00359-4

13. Keys JR, Monk JA, Woolley KL. Sharing Results with Clinical Trial Participants: Insights from an Online Survey of Chinese Consumers. Chin Med J (Engl) 2016 Apr 20;129(8):1007–8. doi: https://dx.doi.org/10.4103/0366-6999.179787

14. Lucas PJ, Allnock D, Jessiman T. How are European birth-cohort studies engaging and consulting with young cohort members? BMC Med Res Methodol 2013;13(1):56. doi: 10.1186/1471-2288-13-56

15. Wallwiener M, Wallwiener CW, Brucker SY, Hartkopf AD, Fehm TN, Kansy JK. The Brustkrebs-Studien.de website for breast cancer patients: User acceptance of a German internet portal offering information on the disease and treatment options, and a clinical trials matching service. 2010 Dec 2;1:663.

16. Hägglund M, Scott Duncan T, Kai-Larsen K, Hedlin G, Krakau I. IntegrIT - Towards Utilizing the Swedish National Health Information Exchange Platform for Clinical Research. Stud Health Technol Inform 2017;235:146–150.

17. Long CR, Purvis RS, Flood-Grady E, Kimminau KS, Rhyne RL, Burge MR, Stewart MK, Jenkins AJ, James LP, McElfish PA. Health researchers’ experiences, perceptions and barriers related to sharing study results with participants. Health Res Policy Syst 2019;17(1):25. doi: 10.1186/s12961-019-0422-5

18. Abel GA, Cronin AM, Earles K, Gray SW. Accessibility and Quality of Online Cancer-Related Clinical Trial Information for Naive Searchers. Cancer Epidemiol Biomarkers Prev 2015 Oct;24(10):1629–31. doi: https://dx.doi.org/10.1158/1055-9965.EPI-15-0274
